# Assessment of Liver Stiffness in Pediatric Fontan Patients Using Transient Elastography

**DOI:** 10.1155/2016/7125193

**Published:** 2016-08-30

**Authors:** Becky Chen, Richard A. Schreiber, Derek G. Human, James E. Potts, Orlee R. Guttman

**Affiliations:** ^1^Department of Pediatrics, University of British Columbia, Vancouver, BC, Canada; ^2^Division of Gastroenterology, Hepatology and Nutrition, British Columbia Children's Hospital, Vancouver, BC, Canada; ^3^Children's Heart Centre, British Columbia Children's Hospital, Vancouver, BC, Canada

## Abstract

*Background*. Hepatic fibrosis is a potential complication following Fontan surgery and heralds long-term risk for cirrhosis. Transient elastography (TE) is a rapid, noninvasive method to assess liver fibrosis by measuring liver stiffness.* Objectives*. To compare liver stiffness and liver biochemistries in pediatric Fontan patients with age- and sex-matched controls and to determine patients' acceptance of TE.* Methods*. Patients were recruited from British Columbia Children's Hospital. Twenty-two Fontan patients (15 males) were identified. Demographic information and cardiac data were collected. TE was measured using size-appropriate probes.* Results*. The median age of the Fontan cohort was 13.7 (5.9–16.8) years. Time from Fontan surgery to TE was 9.6 (1.0–12.9) years. The median Fontan circuit pressure was 13 (11–14) mmHg. TE values were higher in Fontan patients versus controls (18.6 versus 4.7 kPa, *p* < 0.001). There was no association between TE values and patient age (*r* = 0.41, *p* = 0.058), time since Fontan surgery (*r* = 0.40, *p* = 0.062), or median Fontan circuit pressure (CVP) (*r* = 0.35, *p* = 0.111). Patients found TE to be nonpainful, convenient, and safe.* Conclusions*. TE is feasible to assess liver stiffness in children following Fontan surgery. Pediatric Fontan patients have markedly elevated liver stiffness values. TE may have important utility in liver care follow-up of pediatric Fontan patients.

## 1. Introduction

The Fontan procedure was introduced in 1971 as a long-term palliation for patients with tricuspid atresia, creating a direct systemic venous to pulmonary artery connection [1]. Since then, the procedure has evolved to become the preferred surgical strategy for a wide variety of congenital heart lesions characterized by the presence of a functional single ventricle. A recent series of over 1000 cases from a single institution describes a 95% survival to 10 years in the most recent era, highlighting the fact that large numbers of these patients with complex cardiovascular pathology are now living longer [2].

Hepatic fibrosis and cirrhosis have emerged as important complications after Fontan surgery, particularly given the growing number of long-term survivors of the procedure. Several factors place the liver at risk in these patients. Marked hypoxia and cardiovascular collapse may contribute to hepatic injury at initial presentation, prior to surgery. Ischemic insult to the liver may occur during the Fontan procedure itself. In the postoperative period chronic elevation in systemic venous pressure and decreased cardiac output of the Fontan circulation may result in congestive hepatopathy and hypoxic stress, both of which may lead to chronic liver injury and fibrosis [3].

While the potential for hepatic complications after Fontan surgery is gaining increasing recognition, evaluation of the liver has not yet become standard care for these patients. This is largely because of the limitations associated with liver biopsy, long considered to be the gold standard method for grading the degree of liver fibrosis to determine disease severity and prognosis. Liver biopsy is an invasive procedure with important morbidity and mortality risk, particularly in those patients who require repeated assessments [4]. Moreover, a needle liver biopsy is subject to sampling error, as it assesses only 1/50,000 of the organ, and to considerable intra- and interobserver variation [5–9]. Liver biopsies are especially ill-suited for children because the procedure requires general anesthesia and often hospital admission; parents are often reluctant to consent to it.

There is a need for an alternative noninvasive and reliable method to assess the extent of hepatic fibrosis in Fontan patients as a measure of liver disease severity. Routine laboratory investigations are not sensitive enough to identify patients with mild to moderate fibrosis and imaging techniques including ultrasound, computed tomography, and magnetic resonance imaging are only able to detect hepatic parenchymal changes when the liver fibrosis is already advanced [10].

Recently, transient elastography (TE) has emerged as a valuable tool for evaluating hepatic fibrosis in a variety of conditions including chronic viral hepatitis and nonalcoholic fatty liver disease [11]. TE is a noninvasive medical device that rapidly assesses liver fibrosis by measuring liver stiffness (LS) [12]. TE has been shown to be a highly reproducible and reliable method to assess the degree of hepatic fibrosis in adult patients with chronic liver disease and it has high inter- and intraobserver agreement [13]. An LS score is representative of a much larger sample of liver parenchyma compared to a biopsy, since it is derived from a liver volume that is at least 100 times that of a biopsy sample [14]. TE has been validated in children and even young infants with liver disease [15].

Few studies have assessed LS in pediatric Fontan patients using TE [16, 17]. Our study aimed to compare TE measurements and liver biochemistries in pediatric Fontan patients and healthy age- and sex-matched controls and to explore any association between the LS score and hepatic laboratory values and measures of cardiac function in the Fontan cohort. A secondary aim was to determine how patients perceived the TE experience.

## 2. Methods

### 2.1. Patients

Fontan patients aged less than 18 years were recruited from the Cardiology Clinic at British Columbia Children's Hospital (BCCH) from April 2012 to October 2014. Fontan patients with a coexisting primary liver disease (e.g., Alagille syndrome) were excluded. Healthy control patients without known liver disease were recruited from the Gastroenterology Clinic at BCCH from May to August 2012. Patients were excluded if they weighed less than 3 kg or had a body mass index exceeding the 95th percentile. The study was approved by the University of British Columbia Children's and Women's Health Centre of British Columbia Clinical Research Ethics Board.

We collected the following laboratory studies for all patients: hemoglobin, white blood cell count, neutrophil count, platelet count, international normalized ratio (INR), alanine aminotransferase (ALT), aspartate aminotransferase (AST), gamma-glutamyltranspeptidase (GGT), alkaline phosphatase (ALP), and albumin. APRI was calculated using age-based upper limits of normal for AST. Any abdominal imaging was reviewed. No patient had undergone a liver biopsy at the time of this study. Evidence of protein-losing enteropathy (PLE) was documented; all patients are routinely evaluated for this per protocol in the Cardiology Clinic.

The most recent echocardiography report (performed within 2 months of TE) was used to gather the following: presence or absence of a fenestration; ventricular type (left, right, or undetermined); atrioventricular valve (AV) regurgitation grade (0/1/2/3); and ejection fraction.

The most recent electrocardiogram was reviewed. Results of the most recent cardiac catheterization were reviewed for mean pressure within the Fontan circuit as well as LV systolic and diastolic pressures.

### 2.2. Transient Elastography

A TE device (Fibroscan®, Echosens SA, Paris, France) equipped with two probes (S and M) was used by 3 trained operators. The probes differed in diameter, impulse power (5 MHz in S probe versus 3.5 MHz in M probe), and measurement depth. Probe selection was based on the patient's thoracic perimeter. Using the S probe, 2 different modes can be selected, with S1 mode for thoracic perimeters ≤ 45 cm (measurement depth 15–40 mm) and S2 mode for thoracic perimeters between 45 cm and 75 cm (measurement depth 20–50 mm). The M probe was used for thoracic perimeters > 75 cm (measurement depth 25–65 mm). During the measurement, the patient was positioned supinely, with his/her right arm in maximal abduction and placed behind the head. The probe was placed on the skin between the ribs over the right lobe of the liver. At least 10 validated measurements were collected for each patient (as recommended by the manufacturer) and the median value of the measurements in kilopascals (kPa) was derived. The liver stiffness value was considered valid if the interquartile range (IQR), a reflection of the variability of all validated measurements, did not exceed 30% of the median values.

After TE measurements, each patient completed a questionnaire on which they rated their TE experience using a 5-point Likert scale for 3 factors: pain, convenience, and perception of safety. The 2 patients too young to read the questionnaire independently completed it with the assistance of their parent.

### 2.3. Statistical Analysis

Frequency tables were created for categorical data. The number (*n*) and percentage (%) are reported. A chi-square test was used to test for group differences. The median value (range) was calculated for all continuous variables. The AST to platelet ratio index (APRI) as an estimate of the severity of liver fibrosis was calculated as follows: [AST (IU/L)/AST upper limit of normal (IU/L)] × 100/platelet count 10^9^/L [18]. A Wilcoxon Rank Sum test was used to compare the two groups. Pearson correlation coefficients were calculated to determine the association between selected variables. The diagnostic accuracy of TE to predict CVP or APRI was measured using receiver operating characteristic curve analysis (ROC). The area under the curve is reported. Values less than 0.60 were considered “worthless”; values between 0.60 and 0.70 were considered “poor”; values between 0.70 and 0.80 were considered “fair”; values between 0.80 and 0.90 were considered “good”; and values between 0.90 and 1.00 were considered “excellent” in terms of diagnostic accuracy. All statistical tests were two-sided with a *p* value of less than 0.05 considered statistically significant. Statistical analyses were completed using SAS Statistical Software version 9.4 (SAS Institute, Cary, NC).

## 3. Results

Twenty-two patients after Fontan operation (15 males) enrolled in the study. One hundred and four healthy controls without liver disease were enrolled from the Gastroenterology Clinic. Twenty-two control patients who were sex- and age-matched within 18 months of age were included in final analysis. The majority of control patients had celiac disease (Table 1). All control patients had normal hepatic biochemistry and no evidence of liver disease based on chart review, parental history, and physical examination. The Fontan and control patients were similar with respect to height, weight, and BMI (Table 2).

The median age of the 22 Fontan patients at the time of TE was 13.7 (5.9–16.8) years. The primary cardiac diagnoses of these patients are listed in Table 3. The median interval from Fontan operation to TE measurement was 9.6 (1.0–12.9) years. None of the Fontan patients had ventricular end-diastolic pressures above 15 mmHg and none were in cardiac failure. Ventricular function was preserved in all Fontan patients and all were in normal sinus rhythm. Two patients had a persisting fenestration and 4 patients were affected by protein-losing enteropathy (PLE). The median Fontan pressure (CVP) was 13 (8–17) mmHg. On physical examination, 9 Fontan patients had hepatomegaly and 2 had splenomegaly. Abdominal imaging was available on only 1 patient, for whom physical examination and imaging were both normal.

Fontan patients had significantly higher serum ALT (31.4 versus 17.0 U/L, *p* = 0.002) and AST (38.5 versus 27.5 U/L, *p* < 0.001) levels compared to control patients, although all values were within the range of normal provided by the respective laboratories (Table 4). GGT was significantly higher in Fontan patients than in control patients (49 versus 10 U/L, resp., *p* < 0.001), with GGT greater than twice the upper limit of normal in 4 of 15 Fontan patients. Fontan patients had lower serum albumin compared to the control group (38 versus 47 g/L, *p* = 0.041). The platelet count was lower in the Fontan cohort (203 versus 272 × 10^9^/L, *p* = 0.003). The median APRI value was higher in the Fontan patients (0.44 versus 0.25, *p* < 0.001).

**Figure 1:**
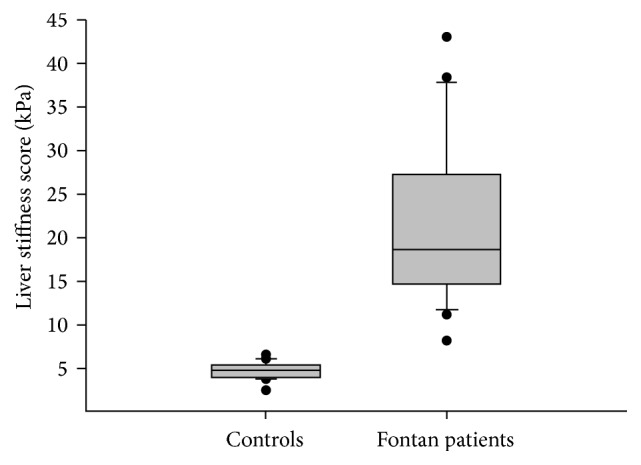
Box plots representing transient elastography results by study cohort. The 10th, 25th, 50th, 75th, and 90th percentiles are reported. Outliers are represented by •.

TE was successfully performed in all study participants. LS values were significantly higher in Fontan patients compared with controls (18.6 versus 4.7 kPa, *p* < 0.001) (Figure 1). LS values of the study control group (*n* = 22) were not significantly different from those of the remainder of the initial group of 105 healthy patients (4.7 versus 4.8 kPa),  *p* = 0.820). The median LS score for the 4 patients with PLE was 27.7 (16.0–42.2) kPa and their median CVP was 13.5 (12–14) mmHg. When these 4 patients were excluded from analysis, the LS values of the remaining Fontan patients remained significantly different versus the control group (17.8 versus 4.7 kPa, *p* < 0.001). The majority of Fontan patients perceived the TE assessment to be nonpainful (83%), very convenient (90%), and very safe (95%). Similarly, most control patients found the assessment to be not painful (77%), very convenient (95%), and very safe (100%).

**Figure 2:**
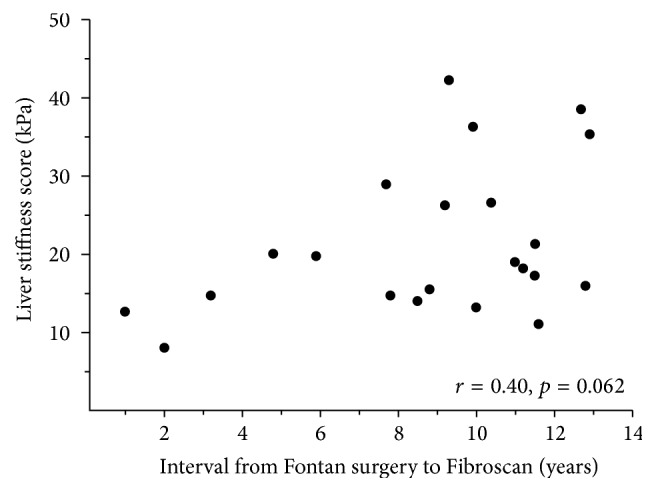
Transient elastography results versus time interval since Fontan.

There was no association between LS and the age at Fontan surgery (*r* = 0.41, *p* = 0.058) or time interval since Fontan surgery (*r* = 0.40, *p* = 0.062) (Figure 2). There was also no association between the LS and mean Fontan circuit pressure (CVP) for the Fontan cohort (*r* = 0.35, *p* = 0.111). In the Fontan cohort, the AUC of LS for identification of CVP >12.5 mmHg was 0.707 and for identification of an APRI <0.46 was 0.703, both reflecting only fair diagnostic accuracy. Median time from cardiac catheterization to TE was 7.0 years (range: 1 day–13.0 years).

## 4. Discussion

In recent years, chronic end-stage liver disease has garnered increasing interest and importance as a long-term complication following Fontan surgery. Early detection for the presence and severity of liver injury is clinically relevant to Fontan patients as it allows assessment of risk for advanced liver disease and its sequelae, as well as potential for early treatment intervention and evaluation [19]. Complications of advanced liver disease and cirrhosis such as portal hypertension with esophageal variceal hemorrhage, ascites, and hepatorenal syndrome portend significant risk to Fontan patients and contribute to their overall morbidity and mortality [20]. Advanced liver injury is also associated with shorter time to sudden death, death from congestive heart failure, and cardiac transplantation [21].

The evaluation of liver fibrosis to determine the severity of liver disease in the Fontan population is challenging, and TE has emerged as a potentially useful tool. TE has been shown to be a reliable and safe noninvasive method to assess liver fibrosis in a variety of adult chronic liver diseases [22]. Data on TE remains limited both in healthy children and in those with liver disease. In pediatric TE studies, it is important to employ S or M probes, to ensure accurate sampling of liver tissue at an appropriate distance from the skin surface based on calculated tissue depth. In this study, TE measurements using size-appropriate S (pediatric) or M (adult) probes found the median LS score in 105 healthy children to be 4.8 (range: 2.4–6.5) kPa. Our results concur with the largest published TE study of 270 healthy pediatric patients reporting normal LS as 4.5 (range: 2.5–8.9) kPa [23]. The interpretation of LS values in children with liver disease is challenging, as most studies thus far have been small and heterogeneous and have mostly used adult-sized M probes to measure the liver stiffness. Indeed, the largest pediatric study of 103 children undergoing liver biopsy for chronic liver disease excluded those patients who were too small for the adult (M) probe [24]. The study reported that TE was a good discriminator of significant fibrosis and suggested that optimal pediatric LS score cutoffs were 6.8 kPa for significant fibrosis (Metavir histologic fibrosis score *F* ≥ 2), 7.5 kPa for severe fibrosis (*F* ≥ 3), and 14.1 kPa for cirrhosis (*F*4). A meta-analysis of studies in adults proposed an LS score of 7.7 kPa as the cutoff value for significant fibrosis and 15.08 kPa for cirrhosis [25]. These results and others suggest that LS cutoff values, as a reflection of the severity of hepatic fibrosis, may differ within pediatric and adult age groups and with disease etiologies [22].

Our study employed size-appropriate TE probes to examine the LS of Fontan patients at a median of 109 months after Fontan procedure. We demonstrated significantly higher LS scores in the post-Fontan group when compared with healthy controls, with the LS scores in a range customarily associated with advanced fibrosis or cirrhosis. A recent study of 45 adult and pediatric patients after Fontan using size-appropriate TE probes found results similar to our own, with a mean liver stiffness of 21.4 ± 10.8 kPa for the cohort as a whole [16]. Using elastic shear wave elastography (a technology similar to TE), Kutty et al. found significantly higher hepatic stiffness compared to controls in a study of 41 adult and pediatric Fontan patients [26]. Our findings are also consistent with those of Friedrich-Rust et al., who evaluated 39 pediatric patients (11.6 ± 5.5 years) with a time interval of 67.9 ± 41.3 months since Fontan operation [27]. While the latter study did not use the pediatric S probe and applied adult cutoff values to predict the extent of liver fibrosis without actual liver biopsy tissue, the authors concluded that 87% of the Fontan patients had LS results suggestive of at least significant liver fibrosis (≥7.2 kPa, *F* ≥ 2) and 41% had results suggesting cirrhosis (≥17.6 kPa, *F*4). Using a similar approach, our results would suggest that 100% of our patients had *F*2 and 58% had *F*4 liver fibrosis.

The use of TE may be limited by a number of clinical variables. It cannot be used in individuals with ascites and is associated with unreliable results in obese patients due to subcutaneous fat interfering with the propagation of the probe signal. Recent data suggest that LS values may be 1.3–3 times higher in acute liver inflammation and moderately elevated ALT [28]. Hepatic venous congestion alone may also increase the liver stiffness yielding an elevated score, even in the absence of fibrosis. This has important implications regarding the interpretation of LS values in Fontan patients. It is likely that LS cutoff values derived from chronic liver disease of other etiologies cannot be directly applied to predict the extent of liver fibrosis in Fontan patients. Indeed, among the 10 Fontan patients who underwent transjugular liver biopsy in the Kutty et al. study, 4 patients with no or minimal fibrosis (<*F*2) still had elevated LS scores of 13.4 ± 1.3 kPa, in a range that would be considered cirrhotic in several other adult liver diseases [26]. While ideally Fontan-specific LS score cutoff values based on hepatic histology assessments will be developed in the future to predict liver fibrosis stage, we recognize that this may not be possible given the confounding influence of congestive hepatopathy on the LS value in these patients. Despite this challenge, we believe TE may still have important utility in this patient population as it may help to identify those with high-risk congestive hepatopathy who might be candidates for treatment to reduce right-sided cardiac pressure.

Biochemical liver function test abnormalities in Fontan patients are typically mild and often show a cholestatic pattern. These have been reported to occur from 2 to 22 years after the Fontan procedure [29–32]. Similar to the findings in this study, the most common reported abnormality is an elevated GGT in 40–60% of patients studied [29–32]. In these patients liver congestion may affect the vascular supply to the intrahepatic bile ducts and cause bile duct epithelial cell injury. While several studies also identified higher ALT levels to about 2-3 times the upper limit of normal in Fontan patients, we did not find ALT or AST elevation above normal in this study, although the absolute values were higher in the Fontan cohort compared to the control group [29–32]. APRI, a model that was developed as a simple, easily calculated method to predict severe fibrosis or cirrhosis, also found higher values in the Fontan cohort (0.44 versus 0.25, *p* < 0.001). In adult patients with hepatitis C, APRI values correlate significantly with the degree of liver fibrosis. APRI values < 0.5 and >1.5 predict the “absence” or “presence” of significant fibrosis (cirrhosis), respectively [33]. While the APRI values in this study, similar to the findings by Yoo et al., were not remarkably elevated above normal in the Fontan group, analysis of the ROC curve in our study showed only “fair” predictive accuracy for the LS score and APRI [17]. This would support the view that liver congestion, in addition to fibrosis, contributes to the LS score observed in the Fontan patients.

In our patient group, the median value and range for CVP were within the expected values for the Fontan population. All of the Fontan patients were in sinus rhythm and had normal ventricular function indicating a satisfactory hemodynamic status. The isolated measure of CVP was not a good predictor of liver stiffness. Analysis of the ROC curve showed only “fair” predictive accuracy for the LS score and CVP, though this may be related to the variability in timing between cardiac catheterization and TE. This is in contrast to the study by Jalal et al. that showed a strong predictive value (AUC = 0.972) of LS for identification of a CVP above 10 mmHg in 60 pediatric patients with a variety of congenital heart lesions [34]. However, in their cohort there were only 2 pediatric Fontan patients and the LS scores in their overall cohort were considerably lower than in our study.

Most of the studies reporting hepatic pathology in Fontan patients show a relationship between the time interval since Fontan and the development of hepatic fibrosis. The Southampton group identified a moderate association between the Fontan duration and the degree of hepatic fibrosis (*r* = 0.75, *p* = 0.013) [35]. A report of 33 postmortem Fontan patients found a correlation between the degree of both sinusoidal and portal fibrosis and the time since the Fontan operation [36]. Another autopsy study found a significant association between time from Fontan and degree of portal fibrosis but no association with sinusoidal fibrosis [22]. Baek et al. found that complications such as cirrhosis, as evidenced by a nodular liver surface on CT, hepatic nodules on imaging studies, hyperbilirubinemia, and thrombocytopenia were significantly associated with length of exposure to the Fontan circulation [37]. Here, the risk of hepatic complications increased as the time since Fontan operation exceeded 10 years (OR = 4.38, CI: 1.11–17.23 for 11–15 years after Fontan) [37].

In view of these data, a recent multidisciplinary symposium has recommended the evaluation of liver histology by liver biopsy in all patients at approximately 10 years after the Fontan surgery [19]. However, our study found no association between liver stiffness and age or time since Fontan operation. This may be the result of our cohort being young, still showing normal hemodynamics rather than having a failing Fontan circuit, and the small sample size. Yet the study by Yoo et al. of 22 adults having Fontan for a mean duration of 13 years did not find a statistically significant correlation between time with Fontan circulation and LS score [17]. A recent study of 19 liver biopsies in 13 Fontan patients (the majority of whom had a failing Fontan) also found no relationship between time interval and extent of fibrosis [38]. Of interest, a large postmortem study evaluated patients who died <35 days postoperatively and found sinusoidal fibrosis in 65% and portal fibrosis in 30%, suggesting that duration of exposure to the Fontan circulation may not be the sole factor in the development of fibrosis.

We believe that further studies are necessary to determine the optimal timing of liver biopsy in this patient population. TE may prove to be an important adjuvant tool in this regard, provided reliable LS cutoff values for fibrosis can be established. Repeated TE testing might also be clinically useful in individual patients as a method to monitor their disease progression, be it advancing fibrosis or worsening congestive hepatopathy. Further evaluation is indicated to elaborate on the role of TE and APRI, when used in conjunction with other modalities, in the discrimination of fibrosis from hepatic congestion in Fontan patients, as well as the potential value of these tools in predicting outcomes (e.g., risk for complications of cirrhosis, liver survival, and need for liver transplantation) in this challenging patient group.

## 5. Conclusion

Hepatic fibrosis and cirrhosis are important potential complications of the Fontan operation that require assessment in this population especially as patients survive longer. Our study has shown that patients after Fontan demonstrate significantly increased LS as well as abnormalities of liver-related laboratory tests compared with healthy age- and sex-matched controls. The hemodynamics of the Fontan circulation in our patient cohort failed to show an association between LS score and age at Fontan, time since Fontan surgery, and CVP. The TE device (Fibroscan) was well accepted by these children, and the device, being noninvasive and safe, may have an important role in the evaluation of the onset and progression of hepatic fibrosis in these patients. TE may also have important utility to identify Fontan patients with high-risk congestive hepatopathy. Further studies involving serial determinations of LS combined with histologic evaluation and measurement of cardiac indices in these cases may help standardize LS scores for this patient population. Protocols to provide liver care should also be considered as adjuvant measures for the post-Fontan patient population.

## Figures and Tables

**Table 1:** Diagnoses of the 22 control patients.

Diagnosis	Number (%)
Celiac disease	11 (50)
Abdominal pain	6 (27)
Nausea	2 (8)
Achalasia	1 (5)
Eosinophilic esophagitis	1 (5)
Gastritis	1 (5)

**Table 2:** Patient demographics.

	Fontan (*n* = 22)	Controls (*n* = 22)	*p*
Age (yr)	13.7 (5.9–16.8)	13.3 (5.7–17.7)	0.963
Height (cm)	148.3 (112.0–191.0)	159.6 (104.3–184.2)	0.250
Weight (kg)	39.9 (17.0–82.2)	42.8 (15.7–72.0)	0.639
BMI (kg/m^2^)	17.8 (13.6–27.0)	17.4 (14.4–27.0)	0.725

BMI: body mass index.

**Table 3:** Primary cardiac diagnoses of the Fontan patients.

Primary cardiac diagnosis	Number of patients (%)
Double inlet left ventricle	4 (18)
Tricuspid atresia	4 (18)
Hypoplastic left heart	3 (14)
Double outlet left ventricle	2 (9)
Univentricular heart	2 (9)
Double outlet right heart	2 (9)
Other	5 (23)

**Table 4:** Laboratory results.

Investigation	Fontan patients	Controls	*p*
ALT (U/L)	31.5 (12.0–54.0)	17.0 (12.0–32.0)	0.02
AST (U/L)	38.5 (17.0–68.0)	27.5 (17.0–36.0)	0.01
ALP (U/L)	231.5 (38.0–520.0)	264.0 (112.0–458.0)	0.33
GGT (U/L)	49 (10–148)	10 (8–48)	<0.001
Albumin (g/L)	38 (22–50)	47 (44–51)	0.041
Platelets (×10^9^/L)	203 (83–376)	272 (183–405)	0.02

ALT: alanine aminotransferase; AST: aspartate aminotransferase; GGT: gamma-glutamyltranspeptidase; ALP: alkaline phosphatase.
